# Genetic Association of HTR1B and HTR2A Gene Polymorphisms with ADHD in Korean Children and Adolescents: A Case Control Study

**DOI:** 10.3390/genes17050546

**Published:** 2026-05-02

**Authors:** Yeongsuk Lee, Hyung Jun Kim, Han Jun Jin, Ho Jang Kwon, Se Hoon Shim, Myung Ho Lim

**Affiliations:** 1 Department of Psychiatry, Graduate School, Soonchunhyang University, Asan 31538, Republic of Korea; 1121lys@hanmail.net; 2 Hyekang Hospital, Cheonan 31122, Republic of Korea; 3 Department of Biological Sciences, College of Science & Technology, Dankook University, Cheonan 31116, Republic of Korea; laons16@gmail.com (H.J.K.); jins4658@dankook.ac.kr (H.J.J.); 4 Department of Preventive Medine, College of Medicine, Dankook University, Cheonan 31116, Republic of Korea; hojang@dku.edu; 5 Department of Psychiatry, College of Medicine, Soonchunhyang University, Asan 31538, Republic of Korea; 6 Department of Psychology and Psychotheray, College of Health Science, Dankook University, Cheonan 31116, Republic of Korea

**Keywords:** attention-deficit hyperactivity disorder (ADHD), serotonin, HTR1B, HTR2A, genetic polymorphism, association study

## Abstract

Objectives: Attention-deficit hyperactivity disorder (ADHD) is the most prevalent neurodevelopmental disorder diagnosed during childhood, primarily characterized by continuous symptoms of inattention, hyperactivity, and impulsivity. The present study aimed to investigate the genetic association between polymorphisms in the serotonergic system-related genes, HTR1B and HTR2A, and the susceptibility to ADHD in a Korean sample. Methods: The study cohort consisted of 234 children diagnosed with ADHD and 1686 healthy controls. Clinical diagnosis was established based on the Diagnostic and Statistical Manual of Mental Disorders, 4th edition (DSM-IV) criteria. Genetic analysis focused on single nucleotide polymorphisms (SNPs) within the serotonergic pathway: rs6296 in HTR1B, and three SNPs (rs6311, rs6313, and rs9534495) in HTR2A. Genotype and allele frequencies were analyzed using Chi-square tests. Risk estimates were calculated as odds ratios (OR) with 95% confidence intervals (CI) across dominant, recessive, and additive inheritance models. Results: A statistically significant association was observed between the HTR2A rs9534495 polymorphism and ADHD. Specifically, significant associations were identified under the dominant (OR 0.67, 95% CI 0.48–0.93, *p* = 0.017), recessive (OR 0.67, 95% CI 0.48–0.93, *p* = 0.016), and additive (OR 0.80, 95% CI 0.65–1.00, *p* = 0.046) models. However, these significant findings did not persist after applying the Bonferroni correction for multiple comparisons. Conversely, no significant associations were detected for the HTR1B (rs6296) and the other HTR2A (rs6311, rs6313) polymorphisms. Conclusions: These findings suggest that genetic variations in the serotonergic system, particularly within the HTR2A gene, may contribute to the genetic susceptibility to ADHD. This study confirmed gene SNIPs associated with the serotonergic system in the pathophysiology of ADHD. Future research involving large-scale multi-ethnic cohorts, functional assays, and gene–environment interaction analyses is warranted to further elucidate the underlying mechanisms of serotonergic genes.

## 1. Introduction

Attention-deficit hyperactivity disorder (ADHD) is the most frequently diagnosed neurodevelopmental disorder in childhood, fundamentally characterized by core symptoms of inattention, hyperactivity, and impulsivity [1,2]. Traditionally, the “catecholaminergic hypothesis,” which emphasizes an imbalance within the dopaminergic system, has served as the predominant explanatory framework for ADHD. However, contemporary research increasingly suggests that the serotonergic system, along with other neurotransmitter pathways, plays a pivotal role in the disorder’s etiology [3]. The serotonergic system interacts extensively with dopaminergic pathways and is essential for the regulation of cognitive functions, motor activity, sleep, pain perception, mood, and aggression [4,5].

A large scale meta-analysis published in 2015, which synthesized data from 179 prevalence studies, reported a global ADHD prevalence of approximately 7.2% [6]. ADHD is recognized as one of the most highly heritable psychiatric disorders; quantitative genetic studies estimate its heritability to be between 60% and 90% [7]. Within the realm of ADHD genetics, the candidate gene approach focuses on specific loci hypothesized to be involved in the pathophysiology of the disorder. While initial research primarily focused on dopaminergic genes (e.g., DRD4, and DRD5), recent scholarship has underscored the significance of serotonergic genes, highlighting their broad interactions with the dopaminergic system and their critical role in behavioral regulation [8].

The HTR1B gene encodes the 5-HT1B receptor and stands as one of the most consistently implicated serotonergic genes in ADHD genetic studies [8]. The 5-HT1B receptor encoded by HTR1B is a Gαi/o-coupled receptor that, upon activation, inhibits adenylate cyclase and reduces intracellular cAMP. At presynaptic serotonergic nerve terminal, it functions as an autoreceptor that suppresses 5-HT release via Gβγ-mediated inhibition of voltage-gated calcium channels and activation of inwardly rectifying potassium (GIRK) channels [9]. Cloned in the early 1990s, animal models have demonstrated that this receptor is crucial for modulating behaviors linked to core ADHD symptoms, including motor activity, aggression, impulsivity, and reward processing [8]. The 5-HT1B receptor is a G protein-coupled receptor primarily localized at presynaptic terminals, where it functions as a heteroreceptor regulating the release of various neurotransmitters, including dopamine, glutamate, and acetylcholine [8]. By positively modulating the functional activity of the mesotelencephalic dopaminergic system, the serotonin–dopamine interaction represents a key mechanism supporting the relevance of HTR1B in ADHD pathophysiology [8].

The 5-HT1B receptor is widely distributed across brain regions associated with ADHD, such as the basal ganglia, striatum, nucleus accumbens, and prefrontal cortex [8]. HTR1B knockout mice exhibit ADHD-like behaviors, including increased hyperactivity, impulsivity, and aggression, suggesting that impaired HTR1B function may be linked to the ADHD phenotype [8]. The most extensively studied polymorphism in HTR1B is the 861G>C (rs6296) SNP. Although this variant is located within the coding region, it is a synonymous substitution; nevertheless, it is hypothesized to influence receptor expression levels by affecting mRNA stability, splicing, or translation efficiency [8]. A seminal study by Hawi et al. [10], using a multicenter European sample of 273 nuclear families, found a significant preferential transmission of the 861G allele to affected offspring, providing early evidence for the serotonergic system’s role in ADHD development [10]. Furthermore, a meta-analysis by Hou et al. [8] confirmed that the HTR1B rs6296 861G allele significantly increases the risk of ADHD (OR = 1.09) [8].

The HTR2A gene encodes the 5-HT2A receptor and has been a primary candidate in ADHD genetic research since its inception [11]. The 5-HT2A receptor encoded by HTR2A is predominantly postsynaptic, with its highest cortical density on the apical dendrites of layer V pyramidal neurons, where it couples to Gαq/11 and modulates glutamatergic excitatory output through phospholipase Cβ–IP_3_/DAG–PKC signaling. A smaller presynaptic pool on cortical glutamatergic terminals and GABAergic interneurons has also been reported but constitutes a minor fraction of total cortical 5-HT2A expression [12]. The 5-HT2A receptor is a Gq/11 protein-coupled receptor that activates the phospholipase C pathway, thereby increasing intracellular calcium levels and activating protein kinase C [11]. This receptor is highly expressed in the cerebral cortex, particularly on the pyramidal neurons of the prefrontal cortex, and plays a vital role in core cognitive domains affected by ADHD, including executive function, attention, and working memory [11].

The most widely researched polymorphisms in the HTR2A gene are the His452Tyr (rs6314) SNP and the -1438A>G (or T102C, rs6311) SNP in the promoter region [11]. The His452Tyr variant, located in the intracellular C-terminal region of the receptor protein, alters the amino acid sequence and potentially affects receptor function [11]. The -1438A>G variant in the promoter region is thought to modulate gene expression [11]. Quist et al. (2000) analyzed the HTR2A His452Tyr and T102C polymorphisms in 115 families with 143 children with ADHD [13]. Their Transmission Disequilibrium Test (TDT) analysis revealed a preferential transmission of the 452Tyr allele to affected offspring (*p* = 0.03), indicating linkage disequilibrium with ADHD [13]. Similarly, Paula et al. (2006) analyzed HTR2A in 243 school aged children with ADHD and their biological parents [14]. While the -1438A>G promoter polymorphism showed no significant association, the His452Tyr polymorphism was linked to ADHD, specifically showing an association with the 452His allele in males (*p* = 0.04).

Based on previous research on the genetic mechanisms of ADHD, we selected four candidate polymorphisms (rs6296 in HTR1B and rs6311, rs6313, and rs9534495 in HTR2A) that had the most previous studies involving the serotonergic system [8,10,13,14]. The objective of the present study is to investigate the association between genotypes and alleles of the HTR1B and HTR2A genes in a sample of Korean children and adolescents with ADHD compared to a healthy control group.

## 2. Methods

### 2.1. Study Population

Patients with ADHD were clinically evaluated by a psychiatrist within one week of their initial visit. Diagnosis was established through clinical interviews and epidemiological assessments based on the criteria defined in the Diagnostic and Statistical Manual of Mental Disorders, 5th Edition (DSM-5). Additionally, a score of 18 or higher on the Korean ADHD Rating Scale (K-ADHD) was required for inclusion in the ADHD group. Exclusion criteria included comorbid intellectual disability, organic brain syndrome, major depressive disorder, or psychotic disorders. The final clinical cohort consisted of 234 patients with ADHD. The control group (n = 1686) comprised children and adolescents with no reported neuropsychiatric disorders, recruited from the general population across 10 cities in South Korea.

All participants and their parents or legal guardians received a comprehensive explanation of the study objectives and provided written informed consent. This study was conducted with the approval of the Institutional Review Board (IRB) of Dankook University Hospital (Ref. No. 2016-08-002).

The demographic characteristics of the participants are summarized in Table 1.

The genetic characteristics of the polymorphisms analyzed in this study are detailed in Table 2.

All chromosomal coordinates refer to GRCh37/hg19. Position was confirmed against dbSNP for rs6296, rs9534495, rs6313, and rs6311.

### 2.2. Experimental Procedures

#### 2.2.1. Epidemiological Assessment

The epidemiological survey included baseline variables such as sex, age, and history of ADHD. Data were extracted from outpatient clinical records, representing the clinical information documented by the attending physicians during patient interviews.

#### 2.2.2. Genotyping

Venous blood samples were collected at the Department of Laboratory Medicine. Genomic DNA was extracted from peripheral blood leukocytes using the Wizard Genomic DNA Purification Kit (Promega, Madison, WI, USA) according to the manufacturer’s instructions. The selected candidate genes included HTR1B and HTR2A. Specifically, the following loci were analyzed: rs6296 for HTR1B, and rs6311, rs6313, and rs9534495 for HTR2A. Genotyping was performed according to our previously established protocols [15]: Genotyping was performed at Macrogen^®^ (Seoul, Republic of Korea) using the Illumina VeraCode 96-plex platform in May 2015 [16]. SNP positions are reported against the GRCh37/hg19 reference assembly, as recorded in the original PLINK.map file supplied by the genotyping facility and verified against dbSNP build 156 [16]. genomic DNA was immobilized on streptavidin-coated magnetic beads, followed by oligonucleotide extension, ligation, and PCR amplification. PCR product preparation, array hybridization, and imaging were outsourced to Macrogen^®^ Inc. (Seoul, Republic of Korea) [16]. Genotypes were determined using Illumina’s proprietary algorithms, which provided quality scores for each call [16].

#### 2.2.3. Statistical Analysis

**Figure 1:**
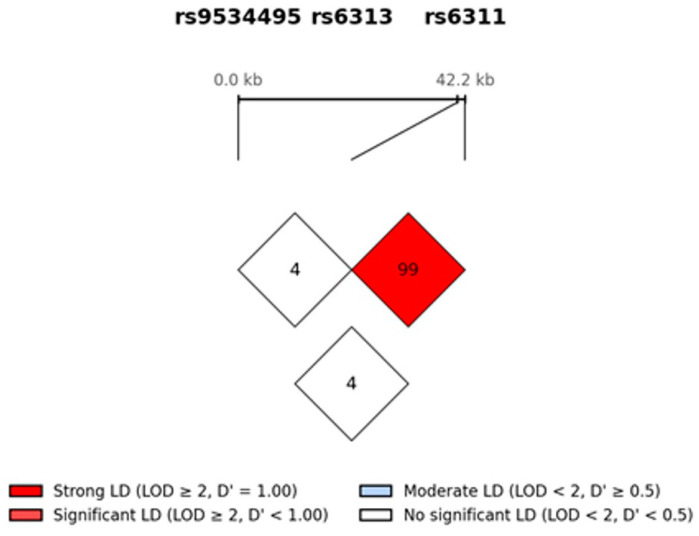
**Linkage Disequilibrium (LD) plot for SNPs in HTR2A genes.** Note: Numbers represent the LD between SNP markers based on D’ values. HTR2A: Serotonin receptor 2A gene. Pairwise LD plot for the three HTR2A SNPs. D′ was computed as |D|/D_max, D′ ranges from 0 (linkage equilibrium) to 1 (complete LD). LOD was computed as log_10_ [L($\hat{D}$)/L(D = 0)]. Numbers within each diamond represent D′ × 100. rs6313–rs6311 are in strong LD (D′ = 0.992, r^2^ = 0.982); rs9534495 is in linkage equilibrium with both downstream SNPs (D′ ≈ 0.04, r^2^ ≈ 0.002). Diamonds show D′ × 100 for each SNP pair; cell color follows the Haploview default scheme—bright red, LOD ≥ 2 and D′ = 1.00 (strong LD); pink, LOD ≥ 2 and D′ < 1.00; light blue, LOD < 2 and D′ ≥ 0.5; white, LOD < 2 and D′ < 0.5 (no significant LD). rs6313 and rs6311 are in near-complete LD (D′ = 0.992, r^2^ = 0.982; LOD ≫ 2), whereas rs9534495 is in linkage equilibrium with both downstream SNPs (D′ = 0.042 and 0.043; r^2^ = 0.002; LOD < 2). HTR1B (rs6296) is on chromosome 6 and is therefore not included in this intra-gene LD assessment; D′ and r^2^ agreed to three decimal places. LD estimated by Python 3.13 EM and cross-validated with Haploview v4.2 (GRCh37/hg19; N = 1828).

Statistical analyses were performed using SPSS version 22.0 (IBM Corp., Armonk, NY, USA). Chi-square tests were employed to compare categorical variables (e.g., sex) and genotype frequencies between groups. Independent *t*-tests were used for age comparisons between the ADHD and control groups. A *p*-value < 0.05 was considered statistically significant. We also performed a Bonferroni correction to correct for errors due to the multiple testing method. Genotype frequencies were further compared between the case and control groups using the web-based software SNPstats (http://www.snpstats.net accessed on 31 May 2026)), with adjustments for age and sex. Hardy–Weinberg Equilibrium (HWE) was verified for each SNP. Additionally, Linkage Disequilibrium (LD) patterns between the genetic polymorphisms were analyzed using the Haploview software 4.2. LOD ≥ 2 was treated as the threshold for statistically significant evidence of LD (Figure 1).

## 3. Results

The association analysis for the HTR2A rs9534495 polymorphism revealed significant differences between the ADHD and control groups. Specifically, significant associations were observed in overall genotype frequency (OR = 0.80, 95% CI = 0.65–1.00, *p* = 0.046). Furthermore, significant associations were identified under the dominant (OR = 0.67, 95% CI = 0.48–0.93, *p* = 0.017), recessive (OR = 0.67, 95% CI = 0.48–0.93, *p* = 0.0167), and additive (OR = 0.80, 95% CI = 0.65–1.00, *p* = 0.046) inheritance models. However, these statistically significant findings did not persist after applying the Bonferroni correction for multiple comparisons (Table 3).

## 4. Discussion

In the present study, the HTR1B rs6296 polymorphism did not exhibit a statistically significant association with ADHD. This finding diverges from several landmark studies; for instance, Hawi et al. (2002) identified a significant preferential transmission of the 861G allele (rs6296) in 273 European nuclear families [9]. Furthermore, meta-analyses conducted by Gizer et al. (2009) and Hou et al. (2018) confirmed a significant linkage between HTR1B and childhood ADHD [8,17].

Conversely, our results align with other research that reported null findings. Ickowicz et al. (2007) failed to find an association between HTR1B SNPs or haplotypes and ADHD in 203 families [18]. Similarly, Heiser et al. (2007) reported no significant association in a German sample [19], and a meta-analysis by Forero et al. (2009) found no evidence of paternal over-transmission of the HTR1B rs6296 allele [20]. The lack of significance in our study provides further support for these negative findings within the literature.

Regarding the HTR2A gene, previous studies have yielded conflicting results. Supporting our potential associations, Quist et al. (2000) reported the preferential transmission of the 452Tyr allele in the HTR2A His452Tyr polymorphism [13]. Paula et al. (2006) also found that the His452Tyr polymorphism was associated with ADHD in a Brazilian sample, particularly noting the link with the 452His allele in males [14]. Furthermore, a study on a Korean population by Cho et al. (2012) identified significant differences in genotype frequencies for the T102C polymorphism [21].

However, other researchers have reported negative outcomes. Hawi et al. (2002) found no association for HTR2A His452Tyr in their total sample, observing significant over-transmission of the 452His allele only within the Irish subgroup (χ^2^ = 4.9, *p* = 0.026) [10]. Heiser et al. (2007) also failed to detect associations for the His452Tyr and -1438G>A variants [19]. Similarly, Güney et al. (2014) reported that the T102C and -1438G/A polymorphisms did not support a primary role for the serotonergic system in the development or clinical course of ADHD [22].

Because rs9534495—which was analyzed in this study—is located within an intron, the observed association may be due to linkage disequilibrium (LD) with rs6313, which can affect protein structure or activity, or with rs6311, which regulates gene expression levels. Furthermore, recent studies have reported that intronic sequence variations can also finely modulate gene expression through mechanisms such as expression quantitative trait loci (eQTLs) or alternative splicing. Meanwhile, the fact that we did not directly analyze rs6314, which has shown the most consistent associations in previous studies, may also be a reason why certain associations were not detected.

The polymorphism analyzed in this study, rs9534495, is distinct from the more frequently studied His452Tyr (rs6314) variant. Since rs9534495 is located in a different region of the HTR2A gene, it may exist in linkage disequilibrium (LD) with rs6314. The fact that we did not directly analyze rs6314 might have limited our ability to detect specific associations.

Discrepancies in HTR2A research may be attributed to several factors. First, the specific polymorphism analyzed is critical. His452Tyr (rs6314) is a non-synonymous substitution that alters the amino acid sequence of the receptor protein, suggesting a higher likelihood of functional impact [13]. In contrast, while T102C (rs6311) is located in the promoter region and may influence gene expression, its functional significance remains less clear [13]. Second, ethnic heterogeneity likely plays a role; for instance, Hawi et al. (2002) noted that HTR2A associations were specific to their Irish sample [10], indicating that the genetic effects of HTR2A may vary across ethnic backgrounds. Given that HTR2A is highly expressed in the prefrontal cortex and is vital for executive functions and attention, its role in ADHD pathophysiology remains biologically plausible, despite the lack of statistical significance in the current study.

This study has several limitations that warrant consideration:

First, although the total sample size was relatively large, the ADHD cohort (N = 311) may have provided insufficient power to detect small effect sizes. In particular, when accounting for multiple comparisons, few associations remained significant after Bonferroni correction. Additionally, we have not looked at all SNIPs related to the serotonergic system. For example, rs6314 was included at the beginning of the methodology but was excluded due to software readout errors. Second, allele frequencies and LD patterns in HTR1B and HTR2A vary significantly across ethnicities; for example, Li et al. (2005) found no association with overall ADHD in a Han Chinese sample but observed a trend toward 861G over-transmission in the inattentive subtype [23]. Third, the case–control design utilized here is more susceptible to population stratification compared to family-based designs (e.g., TDT, FBAT). Fourth, environmental factors such as prenatal complications and psychosocial stress are crucial in ADHD risk. Vega et al. (2023) demonstrated that the association of the HTR1B G-haplotype with ADHD was strengthened when interacting with DRD2, DRD4, and SLC6A4 [24]. The absence of G × E or gene–gene interaction analysis in our study may have masked certain genetic effects.

## 5. Conclusions

In conclusion, our findings suggest that polymorphisms in the HTR2A gene may contribute to genetic susceptibility to ADHD, supporting the hypothesis that the serotonergic system plays a critical role alongside the dopaminergic system in its pathophysiology. However, the genetic architecture of ADHD is remarkably complex and heterogeneous, involving the interplay of multiple genes, environmental factors, and epigenetic mechanisms. Future large-scale, multi-ethnic studies incorporating functional assays and G × E interaction analyses will be essential to further elucidate the complex etiology of ADHD and to develop personalized prevention and treatment strategies.

## Figures and Tables

**Table 1:** Epidemiological characteristics between ADHD group and the control group.

	ADHD Group (n = 234) (m ± sd or n)	Comparison Group (n = 1686) (m ± sd or n)	x2 or t	*p* Value
age ^a^	7.46 ± 1.19	7.46 ± 1.18	<0.01	1.00
sex ^b^			36.97 *	<0.01
men	159 (67.9%)	784 (46.5%)		
women	75 (32.1%)	902 (53.5%)		

These data represent mean ± S.D., by independent *t* test ^a^, or N (%), by chi-square test ^b^, * significant *p* value < 0.05.

**Table 2:** Genomic coordinates and functional annotation of the four genotyped SNPs.

SNP ID	Chromosome: Position	Location	Functional Class	Distance	Alleles	
rs 6296	6: 78,172,260	Coding, synonymous	c.861G>C, p.Val287=	312/860	G/C	HTR1B
rs 6311	13: 47,471,478	Noncoding, promotor	c.-1438A>G, 5′ upstream	−1438	G/A	HTR2A
rs 6313	13: 47,469,940	Coding, synonymous	c.102T>C, p.Ser34=	310/102	C/T	HTR2A
rs 9534495	13: 47,429,228	Noncoding, Intronic	Intron 1	N/A	A/G	HTR2A

**Table 3:** Multivariate model for genotype distribution and allele frequencies of in ADHD group and comparison group.

Gene/SNP rs Number	Alleled: Status	Genotype, n (%)			*p* Value			
		A/A	A/a	a/a	Overall	Dominant model	Recessive model	Additive model
HTR1B/rs 6296	C>G							
	ADHD	66 (21.5)	164 (53.42)	63 (20.52)	0.5	0.206	0.852	0.374
					1.03 (0.85–1.24)	1.22 (0.90–1.65)	0.97 (0.72–1.31)	1.06 (0.89–1.28)
	Controls	330 (21.70)	745 (48.98)	358 (23.54)				
HTR2A/rs 6311	T>C							
	ADHD	73 (23.78)	159 (51.79)	75 (24.43)	0.709	0.427	0.844	0.646
					1.02 (0.86–1.22)	1.12 (0.84–1.49)	0.97 (0.73–1.30)	1.03 (0.87–1.23)
	Controls	368 (24.19)	743 (48.85)	403 (26.50)				
HTR2A/rs 6313	T>C							
	ADHD	73 (23.78)	159 (51.79)	75 (24.43)	0.722	0.408	0.796	0.602
					1.02 (0.86–1.22)	1.13 (0.85–1.50)	0.96 (0.72–1.28)	1.03 (0.87–1.23)
	Controls	372 (24.46)	742 (48.78)	406 (26.69)				
HTR2A/rs 9534495	G>A							
	ADHD	41 (21.35)	88 (45.83)	63 (32.81)	0.046	0.017	0.016	0.046
					0.80 (0.65–1.00)	0.67 (0.48–0.93)	0.67 (0.48–0.93)	0.80 (0.65–1.00)
	Controls	329 (32.04)	695 (67.67)	3 (0.29)				

Note: Data are presented as N (%). Statistical significance was determined by chi-square (χ^2^) test (*p* < 0.05). HTR2A: Serotonin receptor 2A gene; HTR1B: Serotonin receptor 1B gene.

## Data Availability

The data presented in this study are available on request from the corresponding author due to privacy, or ethical reason.
